# Experimental Investigation of Hydraulic Fracturing Fluid Based on Pseudo Gemini Surfactant with Polysaccharide Addition

**DOI:** 10.3390/gels10010030

**Published:** 2023-12-28

**Authors:** Mihail Silin, Lyubov Magadova, Kira Poteshkina, Polina Krisanova, Andrey Filatov, Denis Kryukov

**Affiliations:** Department of Technology of Chemical Substances for the Oil and Gas Industry of Gubkin University, World-Class Research Center «Efficient Development of the Global Liquid Hydrocarbon Reserves», National University of Oil and Gas (Gubkin University), 119991 Moscow, Russia; silin.m@gubkin.ru (M.S.); magadova.l@gubkin.ru (L.M.); poteshkina.k@gubkin.ru (K.P.); denkrukov@yandex.ru (D.K.)

**Keywords:** hydraulic fracturing, gel fracturing fluid, oil and gas production, oil reservoirs, pseudo gemini surfactant, hydroxyethyl cellulose, rheology, proppant suspension, fluid leakoff

## Abstract

In the last decade, hydrogels for hydraulic fracturing based on viscoelastic surfactants have been actively studied. Interest in these systems is justified by their unique qualities: good viscoelasticity and the ability to form stable suspensions of proppant or sand, destruction without the formation of bridging agents, hydrophobization of the rock surface and metal of technological equipment, as well as oil-cleaning properties. These qualities are most often provided by a minimum set of components—a surfactant and an electrolyte. However, the absence of a polymer limits the use of these gels in formations where fluid leakoff is possible. In this article, a liquid was studied, based on a pseudo gemini surfactant (PGVES) with the addition of a water-soluble polysaccharide. The objects of study were selected based on the assumption of interactions between PGVES and the polymer; interactions which favorably influence the technological characteristics of the fracturing fluid. To confirm the hypothesis, rheological studies were carried out. These included rotational viscometry and oscillatory studies at various temperatures. The settling velocity of particles of various proppant fractions was studied and tests were carried out to assess fluid leakoff. The performed experiments show an improvement in the characteristics of the PGVES-based gel under the influence of the polysaccharide. In particular, the rheological properties increase significantly, the stability of proppant suspensions improves, and the fluid leakoff of systems decreases, all of which expands the possibility of using these fracturing fluids and makes this area of experimentation promising.

## 1. Introduction

Currently, in the oil and gas industry, hard-to-recover hydrocarbon reserves belonging to low-permeability, heterogeneous and poorly drained reservoirs are becoming increasingly involved in development. One of the most effective and common methods of increasing production in such wells is hydraulic fracturing. Hydraulic fracturing is a mechanical method of influencing a productive formation and is based on rock rupture along planes of minimum strength, under the excess pressure of fracturing fluid that is pumped into the well. Currently, about a third of hydrocarbon reserves can be recovered only by using this technology [1,2,3,4]. In foreign and Russian practice, aqueous systems based on cross-linked guar gel are most often used as fracturing fluids [5,6,7,8,9,10,11,12]. Cross-linked guar gels are relatively easy to prepare and environmentally friendly. However, problems arise when using guar gum-based compositions. These consist of the significant clogging of the interstitial space with gel residues, which in turn leads to a decrease in permeability [4,13,14,15,16]. In addition, aqueous liquids can negatively affect clay rock, causing it to swell, which also leads to clogging. High viscosity values of cross-linked guar gel provide good stability of proppant suspensions of various sizes; however, due to the high viscosity, short fractures are formed, although the formation of long and branched fractures are desirable [17,18]. For the effective breaking of guar gels, it is necessary to introduce oxidizing or acidic reagents into the composition. Other common fracturing gels are fluids with polyacrylamide additives (also called slickwater). These fluids are as easy to prepare as guar-based fluids. Polyacrylamide-based fluids have lower viscosity values than guar compositions. However, these gels are just as efficient in transporting proppant. There is a problem with the strong adsorption of polyacrylamide and some authors recommend avoiding this by adding urea to the compositions [19]. Polyacrylamide-based gels are much more difficult to break. For this purpose, various peroxide reagents are most often used. Hydrocarbon-based fracturing fluids are currently much less commonly used, although the history of hydraulic fracturing began with these reagents. These gels can be prepared from diesel fuel, gas condensate and commercial oil. Their use is limited because they are harmful to the environment and dangerous to store. Aluminum or iron alkyl phosphates are used for gelling hydrocarbons, while alkaline compounds are used for breaking. In addition to the above, there are also acidic compositions, foam and emulsion compositions that can be used as fracturing fluids [18,20].

For the last decade, as an alternative to classical fracturing fluids, compositions based on viscoelastic solutions of surfactants have begun to develop [14,21,22,23]. Such systems are formed from surfactants with a hydrocarbon radical of 17 or more carbon atoms. They form associates in an aqueous environment in the form of long worm-like micelles. The micelles intertwine with each other and form a network, which leads to the formation of a hydrogel.

Fracturing fluid based on viscoelastic surfactant has a number of positive qualities [17,21,22,23,24,25,26]:

- Viscosity values are lower compared with cross-linked guar gel, which promotes the formation of long and branched fractures;
- High viscoelasticity in fresh and mineralized water, due to which the fracturing has the necessary sand-carrying ability;
- The use of surfactants increases the oil recovery factor;
- Hydrophobization of terrigenous rock and clays, as a result of which the degree of clays’ swelling is reduced, and water blockages do not form after hydraulic fracturing;
- Viscosity reduction when mixed with formation fluids, resulting in complete restoration of rock permeability after hydraulic fracturing (pure fracturing).

One of the new discoveries is pseudo gemini surfactant [14,27,28,29,30,31,32]. They are shown as two surfactant molecules that are represented by higher alkylamines, in the form of which a polybasic acid or its anion can be used. These higher alkylamines are connected to each other by a bridge. PGVES differ from classical dimeric surfactants in that they are formed not by covalent, but by hydrogen and electrostatic bonds between the bridging group and surfactant molecules. This fact determines the ease of obtaining these compounds. There are many studies that seek to examine the properties of pseudo gemini surfactants themselves (their behavior at different pH, the influence of salts, technological properties as fracturing fluids). The behavior of surfactant–polymer mixtures is quite well researched [16,33,34,35,36]. However, today, the behavior of PGVES in a mixture with polymers remains poorly understood. Because there are various functional groups in both the surfactants themselves and the polymers, various intermolecular interactions are quite possible, which can positively affect the properties of these fracturing fluids. Considering that one of the reasons for limiting the use of surfactant-based gels is their tendency toward high fluid leakoff in highly permeable layers [15,16], this research area becomes very relevant, as the polymer will form a certain amount of filter cake. Because the type of bonding in PGVES is very sensitive to the presence of electrolytes, a non-dissociating water-soluble polymer that can interact with PGVES through hydrogen bonds will be chosen as a sample. Based on the above, we can define the purpose of the work: the study of the hydrogel based on PGVES and polymer. To achieve this purpose, it is necessary to carry out rheological and oscillation studies of compositions. Additionally, the technological properties of these gels as hydraulic fracturing fluids will be evaluated. On the basis of the experiments, we will make a conclusion about the promising use of PGVES–polymer hydrogels in hydraulic fracturing and offer recommendations for further research.

## 2. Results and Discussion 

### 2.1. Effective Viscosity Study

To process the obtained values of effective viscosity in a wide range of speeds, the Carreau fluid model was used [37,38], which is expressed by the following formula:

(1) $$\eta -\eta_{\infty }=\frac{\eta_{0}-\eta_{\infty }}{{\left[1+{\left(\lambda \dot{\gamma }\right)}^{2}\right]}^{m/2}},$$

where η is the non-Newtonian apparent viscosity at any shear rate; $\eta_{0}$ and $\eta_{\infty }$ are the plateau of viscosity values at zero shear rate and infinite shear rate, respectively; $\dot{\gamma }$ is shear rate; λ is the characteristic constant of the Carreau model; and m is the dimensionless rate constant. 

**Figure 1:**
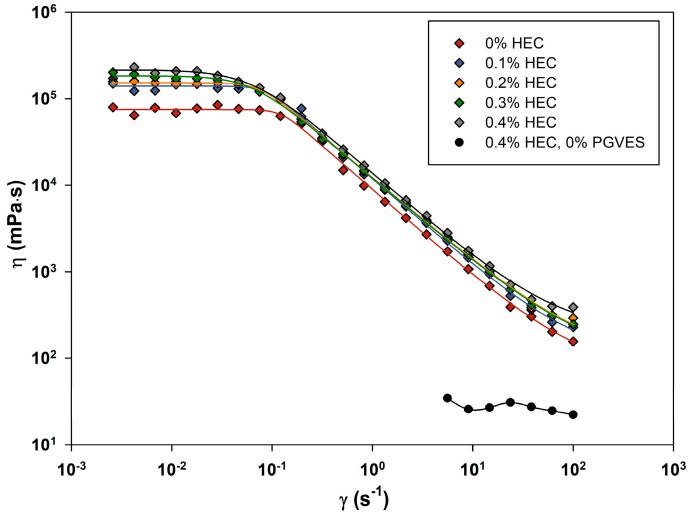
Viscosity versus shear rate for PGVES-based formulations with various hydroxyethyl cellulose (HEC) concentrations at 20 °C.

The results of the experiment at 20 °C are shown in Figure 1. The range of shear rates used is 0.005 s^−1^ to 100 s^−1^.

**Figure 2:**
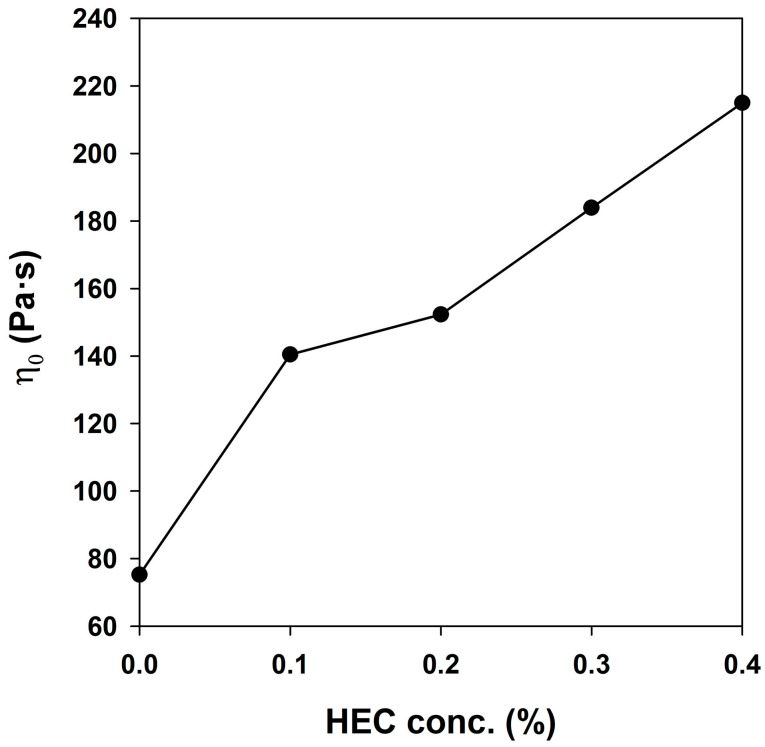
Dependance of zero-shear viscosity on HEC concentration.

At low shear rates, a Newtonian plateau is observed for the systems under study, at which point the viscosity does not depend on the shear rate. For micellar systems, this plateau is explained by the fact that the shear rate imparted to the sample is insufficient to stretch the micelles in the direction of the rotor spinning [32,37]. In the range of the Newtonian plateau, the effect of the HEC additive is most noticeable. Initially, when adding 0.1% HEC, a sharp increase in zero-shear viscosity is observed, and with a further increase in concentration, the increase in viscosity does not occur so intensely, as can be seen in Figure 2. As the shear rate increases, the compositions noticeably lose viscosity. This fact is favorable in relation to fracturing fluid, as its injection is simplified. Separately, we assessed the viscosity of the HEC solution at the maximum concentration (0.4%) without PGVES. Based on the results, we can conclude that there are synergistic effects in the PGVES/HEC system, which may be associated with the formation of surfactant–polymer associates [35,36].

### 2.2. Oscillation Experiments

For the formulations, a linear viscoelastic region (LVER) test was initially performed to find the optimal amplitude value, which was 50%. At this amplitude, the values of the dynamic moduli $G^{\prime }$ (storage modulus) and $G^{\prime \prime }$ (loss modulus) were obtained from the oscillation frequency. The storage modulus $G^{\prime }$ is a parameter that characterizes the elastic properties of a material. This parameter can be used specifically for VES compositions in order to characterize the density of micellar packing and the number of entanglements between micelles. VES compositions are characterized by high values of storage modulus. The high elasticity of the compositions may indicate an optimal mechanical strength of the fracturing fluids, as well as a good stability proppant suspension. Due to the elastic component, when surfactant systems are used as fracturing fluids, a more efficient transfer of the proppant is realized. The elastic component, when surfactant systems are used as fracturing fluids, results in more efficient energy transfer from the wellhead to the bottomhole, which can reduce energy consumption during hydraulic fracturing. Loss modulus $G^{\prime \prime }$ is a parameter that characterizes the behavior of the investigated sample as a viscous fluid. In solutions of worm-like micelles over a wide frequency range, the loss modulus usually has lower values than the storage modulus.

The used frequency range was from 0.01 Hz to 10 Hz (from 0.063 rad/s to 62.832 rad/s). The Maxwell model with one relaxation time is suitable for describing the obtained curves [26,28,39,40,41,42]. According to this model, the dynamic module curves are described by the following equations:

(2) $$G^{\prime }=\frac{G_{0}{\left(\omega \cdot \tau_{R}\right)}^{2}}{1+{\left(\omega \cdot \tau_{R}\right)}^{2}}$$

(3) $$G^{\prime \prime }=\frac{G_{0}\cdot \omega \cdot \tau_{R}}{1+{\left(\omega \cdot \tau_{R}\right)}^{2}}$$

where ω is the oscillation frequency, $\tau_{R}$ is the relaxation time and $G_{0}$ represents the values of the storage modulus on the plateau. Using the intersection point of the dynamic modulus curves $\omega_{c}$, the value of the relaxation time $\tau_{R}\sim 1/\omega_{c}$ can be determined. 

**Figure 3:**
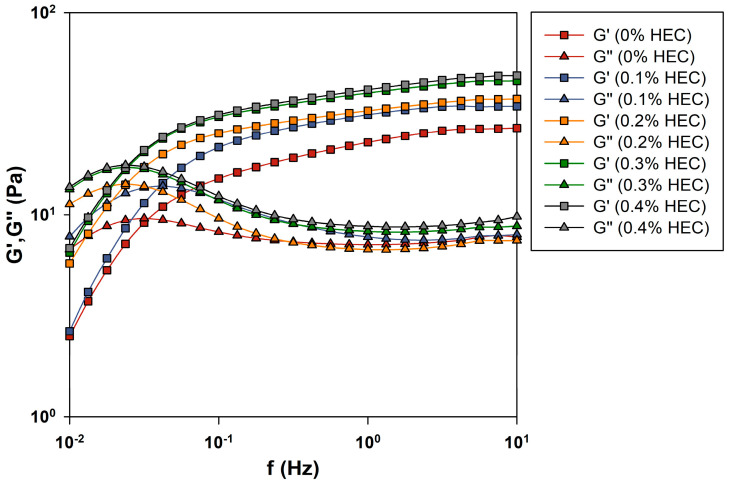
Oscillation studies of PGVES gels with the addition of HEC at 20 °C.

The dependences of the curves of dynamic moduli on the oscillation frequency for gels based on PGVES with HEC are presented in Figure 3. Similar to the values of $\eta_{0}$, the addition of HEC contributes to an increase in the values of dynamic moduli, especially affecting the storage modulus $G^{\prime }$. At low frequencies ($\omega <\omega_{c}$), gels are characterized by the behavior of a viscous liquid; in this range, the values of the loss modulus are greater than the values of the storage modulus. At higher frequencies ($\omega >\omega_{c}$), the storage modulus values become noticeably higher. In this frequency range, the behavior of the gel resembles the behavior of an elastic body. The intersection point of the curves of the dynamic moduli $\omega_{c}$ with increasing polymer concentration moves to lower frequencies, which indicates an increase in the relaxation time $\tau_{R}$. As the relaxation time reflects dynamic processes in the system (the time of rupture and assembly of one micelle), its increase occurs due to compaction of the micellar network with increasing polymer concentration [37,41]. Accordingly, at a HEC concentration of 0.4%, a system with the highest viscoelastic properties is formed.

**Figure 4:**
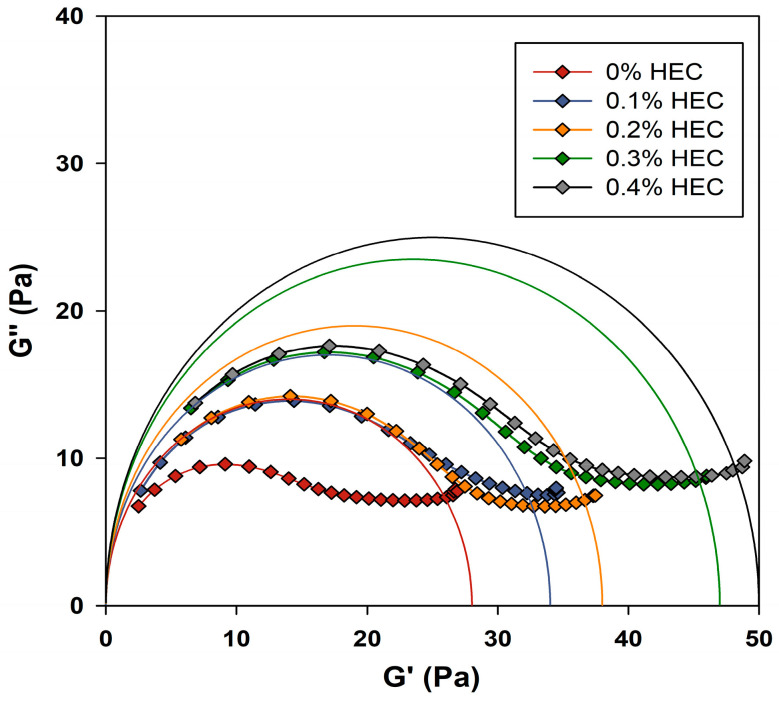
Cole–Cole plots of PGVES gels with HEC additive at 20 °C.

For a more complete study of the mechanism of the HEC influence on the rheological properties of PGVES, one can consider Cole–Cole plots [26,39,42,43], which allow one to evaluate the deviation of the system from the Maxwell model (Figure 4). For each of the compositions, curves were constructed based on theoretical values calculated using the Maxwell model; in the figure they are presented in the form of semicircles and correspond to the following equation:

(4) $${\left(G^{\prime }-\frac{G_{0}}{2}\right)}^{2}+G{\prime \prime }^{2}=\frac{G_{0}^{2}}{4}$$

The experimental data show a strong deviation from the theoretical values of the Maxwell model, which is justified by the large values of the storage modulus [26]. Moreover, this feature can be seen both in compositions with the addition of polymer and in compositions without it. Close intermolecular interactions between PGVES and polymer micelles can contribute to large values of the elastic modulus. An overestimation of the storage modulus values relative to the theoretical values is also observed in the absence of polymer, which indicates intense interactions between PGVES molecules.

Taking into account these data and those obtained from studies of effective viscosity, it can be assumed that the formation of a mixed polymer–micellar system occurs in PGVES–HEC solutions.

### 2.3. Rheological Studies at Elevated Temperatures

**Figure 5:**
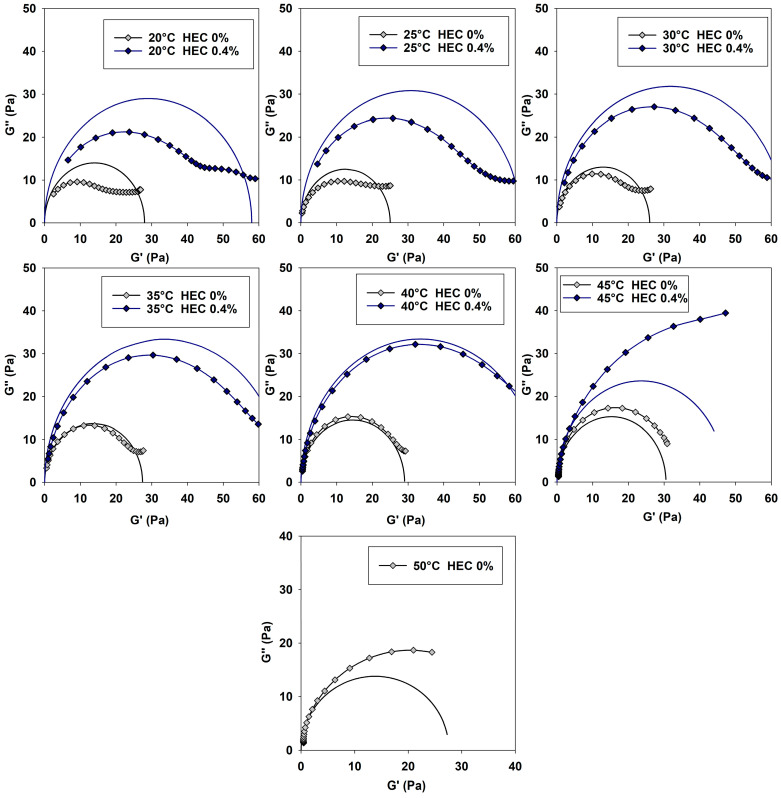
Cole–Cole plots obtained for gels based on PGVES and PGVES with the addition of HEC (0.4%) at temperatures of 20–50 °C.

Micellar systems are formed through intermolecular interactions of various natures. Accordingly, the viscoelastic behavior of such compositions strongly depends on external conditions, especially temperature. Figure 5 shows Cole–Cole plots based on oscillatory data obtained with a gradual increase in temperature. The temperature range was selected from 20 to 50 °C in increments of 5 °C. The studies were carried out for gels based on PGVES and PGVES with the addition of HEC at the maximum concentration (0.4%). 

As the temperature increases, the dynamic processes in micellar systems intensify and at the same time the contour length of the micelles and, as a consequence, the relaxation time decrease [2,4]. The range of oscillatory frequencies at which gels behave like viscous liquids increases with growing temperature. On the Cole–Cole graphs, this is demonstrated by the fact that, as the temperature increases to certain values, the experimental curves become increasingly closer to the curve calculated using the Maxwell model.

**Figure 6:**
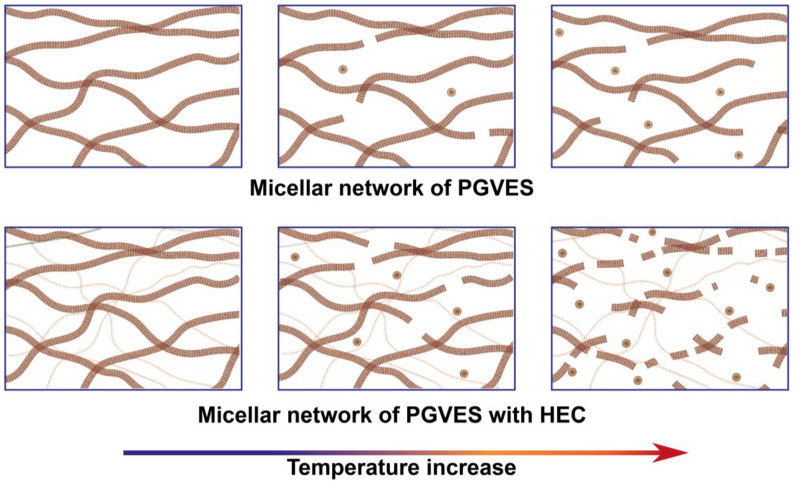
Scheme of degradation of the PGVES micellar network and PGVES with polymer with increasing temperature.

Almost complete equality of the experimental values with the theoretical semicircle for gels based on PGVES and PGVES with HEC occurs at temperatures of 35–40 °C. Moreover, the behavior of compositions with and without a polymer up to 40 °C has a similar trend. A further increase in temperature contributes to the deviation of the experimental values from the theoretical semicircle towards higher values (due to the superiority of the loss modulus over the storage modulus in a wide frequency range). This is mostly distinctive for compositions with polymer. At 50 °C, it is not possible to construct a Cole–Cole plot for a composition with a polymer, as the composition behaves like a viscous liquid over the entire range of oscillation frequencies. A possible explanation for this could be the degradation of hydrogen interactions between PGVES and the polymer with increasing temperature. At high temperatures, polymer molecules act as nothing more than a steric hindrance for PGVES micelles, and the latter, as a result of intense dynamic processes, disintegrate into smaller aggregates [5] (Figure 6). 

Based on the data obtained from oscillatory studies, it is possible to calculate the correlation length of micelles ξ to calculate the distance between the interweaves of the network structure, which characterizes the “density” of the micellar network. The correlation length, as well as other rheological parameters, are presented in Table 1, Table 2 and Table 3. The correlation length of micelles ξ was calculated using the following formula [39,40]:

(5) $$\xi \approx {\left(\frac{k_{B}T}{G_{0}}\right)}^{1/3}$$

where $k_{B}$—Boltzmann constant; T—absolute temperature.

According to the data from Table 1, the injection of HEC helps to increase the density of the micellar network. Note that the correlation length practically does not change with increasing temperature, as shown in Table 2 and Table 3. This is also typical for gels based on PGVES and PGVES with a polymer. However, the values of other rheological parameters, such as relaxation time and zero-shear viscosity, noticeably decrease due to a decrease in the length of the micelles and acceleration of dynamic processes in the gels. 

**Figure 7:**
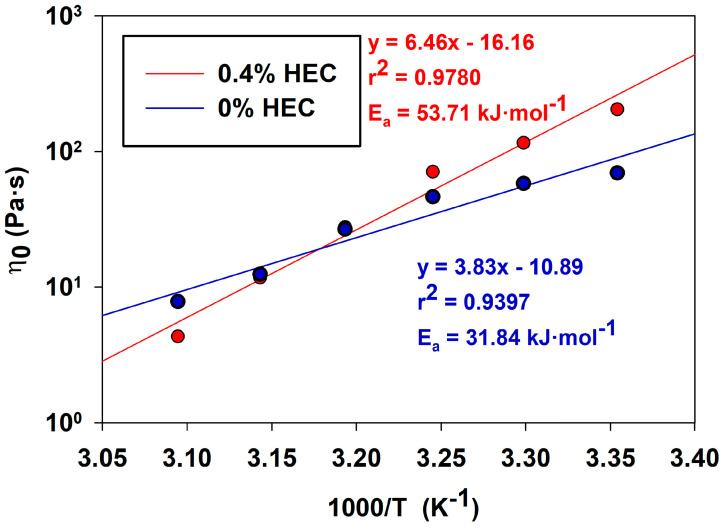
Semi-logarithmic dependence of the zero-shear viscosity of the compositions on the inverse temperature.

Figure 7 shows semi-logarithmic dependences of the zero-shear viscosity of compositions based on PGVES and PGVES with HEC (0.4%) on the reverse temperature. The temperature ranged from 20 to 50 °C. The zero-shear viscosity curve versus temperature is described by the Arrhenius dependence [41,44,45]:

(6) $$\eta_{0}=G_{0}Ae^{E_{a}/RT}$$

where A is the pre-exponential factor, $E_{a}$ is the flow activation energy and R is the universal gas constant.

This dependence arises from the exponentially decreasing length of micelles with increasing temperature. Therefore, the longer the cylindrical micelles, the higher the value of $\eta_{0}$. The experimental values obtained in the work fall in a straight line corresponding to Equation (6). Using the slope of the straight line, we calculated the flow activation energy for the PGVES composition and PGVES with HEC (0.4%).

The calculated values of activation energy $E_{a}$ for compositions based on PGVES and PGVES with 0.4% HEC were 31.84 kJ∙mol^−1^ and 53.71 kJ∙mol^−1^, respectively. For systems consisting of surfactant micelles, the characteristic values of $E_{a}$ lie in the range from 70 to 300 kJ∙mol^−1^ [33,41,44]. The low activation energy values obtained in this experiment indicate the high thermal stability of the compositions. At the same time, for a mixture of PGVES and HEC, the activation energy is higher than for a PGVES solution, because in the latter the drop in viscosity with increasing temperature does not occur so intensely.

### 2.4. Determination of Proppant Particle Settling Velocity

**Figure 8:**
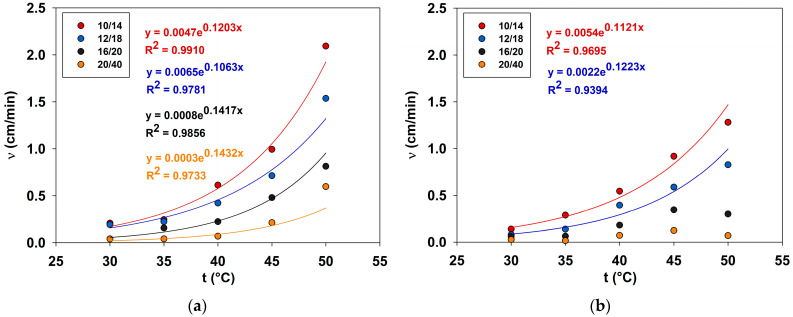
Dependence of the settling velocity of proppant particles on temperature: (**a**) gel based on PGVES; (**b**) gel based on PGVES with the addition of 0.4% HEC.

The proppant settling velocity was determined for four fractions: 10/14, 12/18, 16/20 and 20/40. Particles with maximum sphericity and roundness were selected for the test; to obtain comparable results, the test was carried out on at least five particles. The results of the experiment are presented in Figure 8. 

With increasing temperature, the settling velocity of particles increases, which correlates with the rheological parameters obtained earlier. Experimental points can be described by an exponential curve. However, in the case of a PGVES system with a polymer, the exponential curve can only be used in the case of large proppant fractions: 10/14 and 12/18. The fractions settling velocity of particles 16/20 and 20/40 does not increase exponentially with temperature. In addition, for the gel with the polymer these values are lower than for the gel without, although the rheological parameters with temperature increase degraded more strongly in the polymer gel. It is possible that the values of $G_{0}$, which are much larger in polymer solutions due to a denser micellar network, contribute particularly to this. 

### 2.5. Fluid-Loss 

**Figure 9:**
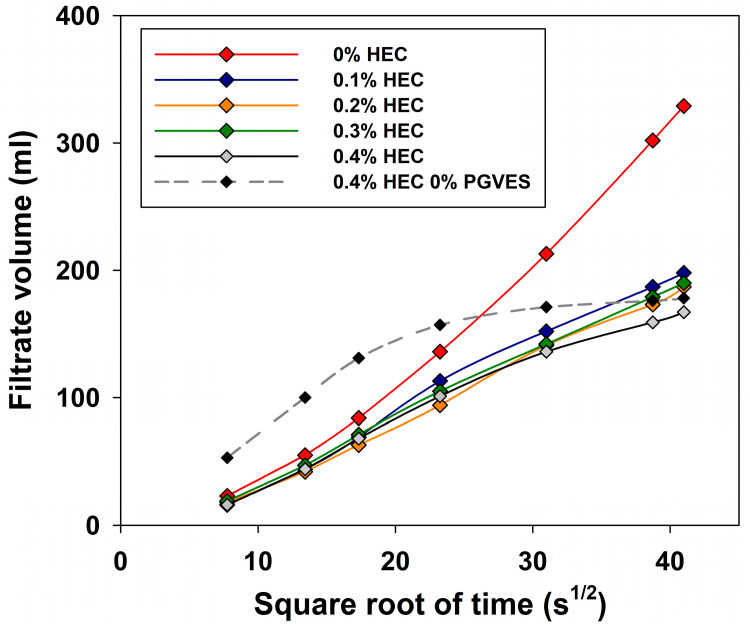
Study of fluid losses of PGVES gels with the addition of HEC.

The results of the study are illustrated in Figure 9.

The experiment revealed a tendency towards a decrease in the rate of fluid losses with increasing polymer concentration, due to the formation of a filter cake on the surface of the filter and higher viscosity of the compositions. Note that a system containing only HEC 0.4% is characterized by a specific form of fluid loss curve with a rapid exit to a plateau due to more intense formation of filter cake [15]. 

## 3. Conclusions 

The research revealed that the addition of HEC can help improve the technological properties of fracturing fluid based on PGVES:

- Rotational and oscillatory studies showed a significant increase in viscosity and viscoelastic properties with the introduction of HEC into the compositions. However, with increasing temperature, these properties degrade more strongly in gels with polymer. For further experiments at elevated temperatures, we recommend the use of cationic or anionic polymers that will interact with PGVES by electrostatic mechanism, this will give better thermostability of the gel. The temperature values used in this work correspond to the field conditions in the Urals and Volga regions of the Russian Federation, and the developed compositions are recommended for these locations;
- In the case of gels with the addition of HEC, the storage modulus on the plateau remains consistently large, which indicates a high density of the micellar network;
- Subsequent studies on the settling velocity of proppant show that the settling velocity of proppant at elevated temperatures, especially fine fractions, is lower for a gel with an injected polymer. It can be concluded that the density of the micelle network has a strong influence on this parameter. Based on this experiment, we recommend the use of coarse proppant fractions in gels with polymer addition at temperatures up to 50 °C. However, good sand-carrying capacity can also be achieved at higher temperatures due to turbulence flow;
- The addition of HEC helps to reduce the degree of fluid loss, which was revealed during experiments on a fluid-loss cell. Formation damage experiments are recommended as further studies.

Considering all of the above, studies on the possibility of using HEC in compositions based on PGVES show promising results. This type of gel can be recommended for further research as a fluid for hydraulic fracturing.

## 4. Materials and Methods 

### 4.1. Materials 

To obtain a pseudo gemini surfactant, N-[3-(dimethylamino)propyl]octadec-9-enamide obtained from SIEGU “Petrohim-Servis” JSC, Moscow (Russia) and oxalic acid 99.2% Hunan (China) were used. Hydroxyethyl cellulose, obtained from Polycell CJSC, Vladimir (Russia), was selected as a water-soluble polysaccharide. Fresh water was used as a base for fracturing gels.

### 4.2. Preparation of Fracturing Gel

**Figure 10:**
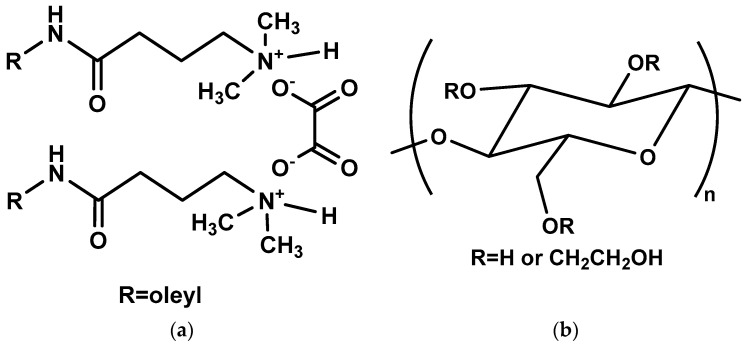
Structural formulas of substances: (**a**) PGVES; (**b**) HEC.

**Figure 11:**
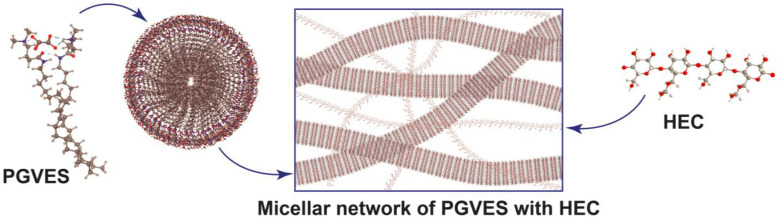
Schematic representations of the structures of PGVES, HEC, and the micelle network.

The concentration of PGVES in all experiments remained constant—6 wt%. The gel was prepared by consistently dissolving calculated amounts of oxalic acid and N-[3-(dimethylamino)propyl]octadec-9-enamide in fresh water at a molar ratio of 1 to 2. When studying gels based on PGVES with HEC, the required amount of polymer was initially dissolved in water, and, after its complete dissolution, reagents were introduced to obtain PGVES. Structural formulas and schematic representations of PGVES and HEC, as well as the resulting micelles, are shown in Figure 10 and Figure 11.

### 4.3. Research Methods

#### 4.3.1. Rotational Viscometry and Oscillatory Rheology 

Rheological studies were carried out on the Anton Paar MCR72 rheometer. A coaxial cylinder measuring system and a double gap measuring system were used. The temperature at the rheometer was controlled using Pelte elements. The experiments were carried out at temperatures from 20 to 50 °C. In rotary mode, the measuring spindle rotates at a constant shear rate and the measuring cup remains stationary. As a result of the experiment, the curves of viscosity dependence on shear rate are obtained. In oscillation mode the measuring system performs oscillatory movements with a given frequency and amplitude. The advantage of this test is the ability to measure the sample without destroying the structure of the sample. When examining a sample in oscillation mode, the first test is always the amplitude sweep test, because this test allows us to determine the linear viscoelasticity range. Knowing this information, we can determine the range of strain values in which the structure of the test specimen does not break under strain. As a result of the oscillation experiment, the curves of the storage modulus and loss modulus dependences on frequency are obtained.

#### 4.3.2. Determination of Proppant Particle Settling Velocity

Ceramic proppant was used in the experiments. The particle diameter ranged from 0.73 to 1.70 mm. To perform the test, the gel was placed in 100 mL cylinders, and the cylinders were placed in a thermostat. After thermostatting the liquid for an hour, proppant particles were introduced into the cylinders and the time it took for the proppant to cover a distance of 1 cm was recorded. Five parallel experiments were carried out at a time and the results were averaged. The settling velocity was assessed at temperatures from 30 to 50 °C.

#### 4.3.3. Experiments on a Fluid-Loss Cell

**Figure 12:**
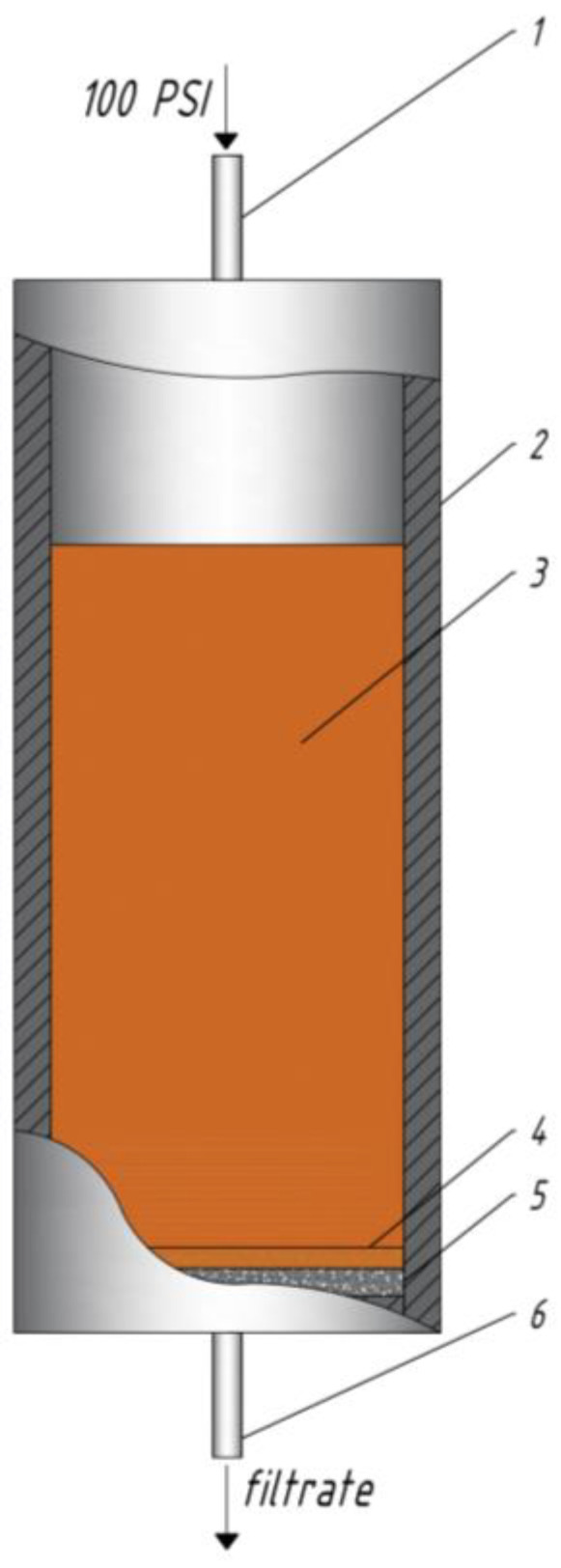
Diagram of a fluid-loss cell: 1—pressurizing valve; 2—fluid-loss cell; 3—test liquid; 4—filter cake; 5—core material or layer of filter paper; 6—filtrate valve.

Fluid-loss cell tests were carried out in accordance with the American standard for fracturing fluids ISO 13503-4 [46]. The gel under study was placed into the cell of the device and a pressure of 100 psi (0.68 MPa) was created. The temperature of the experiments was 25 °C. After holding for 30 min at a given temperature and pressure, the tap is opened and, at certain intervals (1, 3, 5, 9, 16, 25, 30 min), the volume of filtrate flowing into the glass cylinder was measured. The cell diagram is shown in Figure 12. Several layers of filter paper were used as the filtration medium. 

## Figures and Tables

**Table 1:** Changes in the rheological parameters of gels based on PGVES depending on the concentration of HEC at 20 °C.

HEC Concentration (%)	$G_{0}$ (Pa)	$\tau_{R}$ (s)	ξ (nm)	$\eta_{0}$ (Pa)
0	26.72	29.49	53.29	75.20
0.1	34.42	24.86	48.98	140.45
0.2	37.34	41.72	47.67	152.33
0.3	45.94	40.62	44.49	183.93
0.4	48.72	40.50	43.63	214.99

**Table 2:** Changes in rheological parameters of gels based on PGVES depending on temperature.

Temperature (°C)	$G_{0}$ (Pa)	$\tau_{R}$ (s)	ξ (nm)	$\eta_{0}$ (Pa)
20	26.73	29.49	53.29	75.20
25	25.03	8.73	54.78	69.50
30	26.30	11.12	54.19	57.96
35	27.63	8.02	53.59	46.21
40	29.27	4.74	52.85	26.73
45	30.86	1.91	52.20	12.44

**Table 3:** Changes in the rheological parameters of gels based on PGVES with HEC (0.4%) depending on temperature.

Temperature (°C)	$G_{0}$ (Pa)	$\tau_{R}$ (s)	ξ (nm)	$\eta_{0}$ (Pa)
20	48.72	40.50	43.63	214.99
25	53.01	25.41	42.66	204.31
30	54,70	13.48	42.45	115.53
35	56,21	5.78	42.30	70.61
40	58.04	1.33	42.08	27.63
45	47.20	0.37	45.31	11.68

## Data Availability

The data presented in this study are openly available in article.
